# iPads and the Use of “Apps” by Children with Autism Spectrum Disorder: Do They Promote Learning?

**DOI:** 10.3389/fpsyg.2016.01305

**Published:** 2016-08-30

**Authors:** Melissa L. Allen, Calum Hartley, Kate Cain

**Affiliations:** Department of Psychology, Lancaster UniversityLancaster, UK

**Keywords:** Autism Spectrum Disorder, iPad, technology, learning, communication

## Abstract

The advent of electronic tablets, such as Apple's iPad, has opened up the field of learning via technology, and the use of electronic applications (“apps”) on these devices continues to dramatically rise. Children with communication and social impairment, specifically those with Autism Spectrum Disorder (ASD), often use educational and recreational apps within the context of their home and school settings. Here we examine in which contexts learning via this medium may be beneficial, and outline recommendations for the use of electronic tablets and the design features for apps to promote learning in this population that is characterized by a unique profile of needs and heterogeneous ability levels.

## Introduction

Severe language impairments are a common characteristic of Autism Spectrum Disorder (ASD; DSM-V: American Psychiatric Association, 2013). Approximately 80% of children with ASD aged 5 years and younger who enter special education are non-verbal (Bondy and Frost, 1994), and 30% are minimally-verbal at 9-years (Anderson et al., 2007). These linguistic difficulties can have a devastating impact on children's capacity to communicate, but special educators have developed numerous interventions that enable communication without expressive language. In educational and clinical settings, picture-based strategies such as the Picture Exchange Communication System (PECS; Bondy and Frost, 1994) are the most popular due to their low demands, focus on visual spatial processing (a relative strength in ASD; Mottron et al., 1999), and proven capacity to facilitate communication in minimally-verbal children (Flippin et al., 2010). However, the emergence of the Apple iPad in 2010 (and similar tablet hardware) has elicited a surge toward technology-mediated education and interventions, which may benefit children with ASD due to the device's portability, easy to use touch screen interface, and ability to emit multimodal output (Lofland, 2016). While there are countless software applications (“apps”) designed to support language development and communication using digital pictures, little research has investigated the ability of children with ASD to comprehend and learn from symbolic information presented via tablet technology. Similarly, there is little guidance as to how the features of electronic apps can be maximized to specifically facilitate learning in this population. These areas of enquiry are, however, rapidly emerging.

### Symbolic understanding of pictures

Prior to addressing learning via electronic media, it is important to ascertain the extent to which children with ASD understand the symbolic role of pictures, and how they might learn from them. On a fundamental level, many children with ASD have been shown to have a different route of pictorial understanding than typically developing (TD) peers. For instance, Preissler (2008) showed that minimally-verbal children with ASD and cognitive impairment who use picture based systems to communicate associatively mapped words onto black-and-white pictures themselves and failed to extend labels to depicted referents, unlike TD children (see Preissler and Carey, 2004). However, Hartley and Allen (2015a) found that a similar population of children with ASD extended labels to symbolized referents approximately twice as often in color picture trials relative to non-color picture trials (see also Hartley and Allen, 2015b for the facilitative role of iconicity for pictorial comprehension). This finding suggests that different types of pictures may promote or inhibit understanding.

### Perceptual cues: shape and color

Other research shows that when children with ASD generalize names from pictures, they often do so based on atypical cues. In Hartley and Allen (2014a), children with and without ASD learned the names of unfamiliar objects depicted in photographs and were required to sort items according to whether or not they were also referents of the newly-learned names. While the TD controls only generalized labels to items that matched on shape (a category-defining cue), children with ASD frequently generalized to items that matched depicted objects on shape *or* color (a category-irrelevant cue). Thus, it appears that minimally-verbal children with ASD do not know intuitively *what* names refer to when paired with pictures (i.e., the picture itself, the depicted object's shape, or the depicted object's color) and their symbolic comprehension is significantly influenced by the *type* of picture. Taken together, these differences suggest that there might be an atypical route of word learning via pictures in ASD (see also Hartley and Allen, 2014b), but they leave open the question of whether media type (e.g., iPad or book) can impact the capacity for **symbolic understanding**.

KEY CONCEPT 1 **symbolic understanding**

Symbols *represent* external referents in the world, and the relationship between a symbol and its referent is determined by the intention of the creator of the symbol. The word ‘monkey’ and picture of a monkey both refer to, and symbolize, real monkeys. Symbolic understanding of pictures requires that an individual ‘sees through’ the picture to its referent.

### Our study

We thus investigated whether picture-based learning, in particular extension of words learned via pictures to real objects, in children with ASD is impacted by the use of an iPad (Allen M. L. et al., 2015). For TD children, “traditional” picture books facilitate learning because they provide optimal opportunities for joint interaction and engagement (see Ganea et al., 2008). However, by definition, children with ASD are impaired in the domain of social-cognition (DSM-V: American Psychiatric Association, 2013) and are often averse to engaging in social-interactions (Sigman et al., 1986). For this reason, we theorized that the increasingly self-contained nature of the iPad might reduce environmental stress associated with social interaction, allowing greater cognitive resources to be focused on learning. We also explored whether the type of picture impacted children's learning when presented on either the iPad or a more “traditional” picture book.

A critical issue is whether children with ASD show the same biases when developing vocabulary as TD children. Previous research shows that, by 24 months, TD children infer the general rule that noun-referent relations are constrained by shape, and will generalize labels based on this feature rather than other perceptual properties (e.g., color, size, texture; Landau et al., 1988). This “**shape bias**” is driven by TD children's sensitivity to word-shape co-occurrences during infancy (Samuelson and Smith, 2005) and their abstraction of prototypes (mental representations of a category's “central tendency”; Younger, 1990). By contrast, children with ASD do not show an attentional bias for shape in word learning contexts (Tek et al., 2008; Hartley and Allen, 2014a), likely due to deficits in foundational nonverbal processes (Frith and Happé, 1994; Klinger and Dawson, 2001) or a delay in learning the strategy (Field et al., 2016). However, presenting multiple differently-colored examples of a target referent (rather than a single exemplar) when teaching a new name may serve to highlight similarity of shape, thus fostering shape-based generalizations despite unusual attentional biases.

KEY CONCEPT 2 **shape bias**

Children tend to generalize nouns to other category members based upon similarity of shape, rather than other properties of objects such as color, texture, or material. This ‘bias’ helps to explain the rapid and effortless way most typically developing children learn about words and category membership.

To investigate the cues that influence word learning in children with ASD, and whether this is influenced by medium of presentation (e.g., iPad or book), we worked with 16 minimally-verbal children with ASD (*M* receptive language: 3.9 years; *M* nonverbal IQ: 57.5)—the target audience for producers of communication apps on the iPad. All children were recipients of picture-based interventions such as PECS and were frequently exposed to iPads in educational settings. Participants were taught the names of unfamiliar objects presented in photographs across four within-subjects conditions: (1) via an iPad, repeatedly presenting a single representation of the target object, (2) via a picture book, repeatedly presenting a single exemplar, (3) via an iPad, presenting multiple differently colored representations of the target object, and (4) via a picture book, presenting multiple differently colored representations. Children were then tested on their ability to extend the newly-learned names to three-dimensional (3-D) referents matching on shape and color, and to generalize names to novel category members matching on shape but not color. Crucially, our results revealed that medium of presentation—iPad or book—did not impact on children's extension of names from pictures to real objects. Rather, children with ASD only extended labels to depicted objects at above-chance rates when presented with *multiple differently-colored pictures* of the target referent, and tended to map narrow associative word-picture relations when presented with a single exemplar.

By demonstrating that a single label does not refer to a unique referent (i.e., a specific target picture), the multiple example conditions may have increased children's awareness that words can be extended to various items in one's environment, including perceptually similar objects. By contrast, the process of repeatedly pairing a verbal label with one target picture in single exemplar trials may have narrowed the relation to the extent that the picture itself (rather than the depicted object) was more frequently considered the referent of the word (Plaisted, 2001; Preissler, 2008; Hartley and Allen, 2015a). Thus, the nature of the pictures being presented may be a more important influence on symbolic learning in ASD than whether they are presented on an iPad or in a book.

### Do apps benefit communication and learning for children with ASD?

Although our small scale study on word learning did not reveal any advantages in the use of an iPad vs. traditional picture books, other studies report success when teaching communication skills to minimally-verbal children with ASD. Lorah et al. (2015) found that across 17 studies, 93% of individuals improved their ability to communicate by using an iPad or iPod as a multi-functional speech generating device (SGD). Furthermore, they identify several papers that report learning and preference advantages for iPad-based SGDs in comparison to other **augmentative and alternative communication (AAC)** interventions, such as manual sign language and picture exchange protocols (Flores et al., 2012; van der Meer et al., 2012a,b,c; Lorah et al., 2013; Achmadi et al., 2014; Couper et al., 2014). For example, Lorah et al. (2013) revealed that teaching requesting behaviors via an iPad SGD yielded greater overall success, improved maintenance, and required less time in comparison to a “traditional” picture exchange protocol. Another recent study by Xin and Leonard (2015) found that three minimally-verbal children with ASD successfully learned how to initiate requests, respond to questions, and made more frequent social comments after 6 weeks' training on an SGD iPad app (however their study did not include a comparison AAC). Thus, when used as an SGD, the iPad can effectively promote communication in minimally-verbal children with ASD.

KEY CONCEPT 3 **augmentative and alternative communication (AAC)**

AACs encompass a variety of forms of communication to allow an individual with spoken or written language impairment to express their needs. Examples include gestural systems, picture based systems or communication devices with voice output.

However, other studies report that iPad-based interventions are no more effective (and in some respects less effective) than alternative interventions. Agius and Vance (2016) found that three children with ASD mastered a series of requesting behaviors in a similar timeframe when trained on PECS and an iPad-based SGD. Although children achieved similar proportions of independent requesting with both AACs post-intervention, they required fewer prompted responses when learning via PECS (making it more efficient) and follow-up data suggested that maintenance of iPad-supported requesting was reduced. El Zein et al. (2016) compared the effectiveness of a reading comprehension intervention when instruction was teacher-directed or iPad-assisted. While both interventions improved reading comprehension and reduced task refusal, the teacher-directed intervention was relatively more effective at promoting target behaviors.

In a randomized control trial, Fletcher-Watson et al. (2015) examined the efficacy of an iPad-based app targeting social-communication skills in 54 children with ASD below 6 years of age. The game-like app was designed to motivate and rehearse two key joint attention skills—attention to people and social cue following—and was accessed by half of the participants for 2 months (the other half formed a “treatment as usual” control group). The app consisted of two parts. In Part 1, a human character was depicted on the screen and children were required to touch it. Children progressed through increasingly-difficult “levels” that simultaneously presented non-human distractors that had to be ignored. In Part 2, the human character was presented in a shop and pointed toward a desired item at one of six locations around the screen. Children were required to touch the desired item, and the more difficult levels involved the character just looking rather than pointing. The app's efficacy was evaluated by comparing standardized measurements of children's social-communication (e.g., eye contact, quality of social overtures, bids and responses to joint attention) and vocabulary taken before and after the intervention period. Crucially, there were no significant differences between children's pre- and post-intervention scores on any assessment, and time spent playing the app did not correlate with any measured ability. However, the app was highly engaging for children and regarded favorably by parents. These important results call into question the usefulness of iPads for promoting “real world social skills” in children with ASD. However, they do highlight the potential for an intervention administered on a tablet such as the iPad to directly increase levels of engagement, which could be explored in terms of how it might impact upon later learning.

Taken together, these findings suggest that iPad-based interventions can effectively promote certain target skills (e.g., instrumental requests), but not others (e.g., spontaneous social communication). There are also potential differences in the learning mechanisms supporting the two types of skills we reviewed: instrumental requesting relies upon associative learning, whereas spontaneous social communication requires broader social-pragmatic awareness and social motivation which may be more fundamentally impaired in ASD. The balance of evidence suggests that iPads do not readily improve learning and communication for children with ASD, but it is important to note that there is no strong evidence indicating that tablets and educational apps are *detrimental* to learning. ASD presents a unique challenge given the **heterogeneity** of the condition (Folstein and Rosen-Sheidley, 2001), resulting from multifaceted interactions between genes, behavior, and the brain across development (Pelphrey et al., 2011). In addition to differences in language ability, individuals with ASD vary in terms of their cognitive skills (Volkmar et al., 2014), behavioral difficulties, and levels of social understanding (Rice et al., 2012). Due to this variation, learning styles of individuals across the spectrum are not uniform in nature (Tsatsanis, 2004). Tsatsanis (2004) advocates the need for individualized educational programming to directly combat the heterogeneity of learning style and blanket materials often issued within therapeutic intervention for those with ASD. For instance, individuals with and without co-morbid intellectual impairment have differences in memory and attention that affect learning processes. The efficacy of any intervention, whether mediated by technology or teacher/caregiver, depends greatly upon both features of the intervention package itself, as well as the individual child. In the following sections we consider how educational apps used on tablets and iPads have the potential to maximize learning for this heterogeneous population.

KEY CONCEPT 4 **heterogeneity of ASD**

ASD is by definition a spectrum condition, meaning that individuals vary in terms of presentation of core diagnostic behaviors and their severity and levels of adaptive functioning. It is important to consider the vast heterogeneity in diagnosis, research, and treatment, as a singular approach is not sufficient.

### Future research and the potential of language and communication apps for ASD: attitudes to iPad and tablet interventions

An important advantage of iPad-based interventions is that they are often *preferred* over more traditional AACs by children with ASD (Lorah et al., 2013, 2015). This preference may increase the likelihood of children using the app and, through this, elicit greater communication, thereby demonstrating greater learning. In addition to children with ASD showing a preference to use an iPad rather than more traditional AACs, iPads may result in greater engagement and time on task. Research to date supports this: interventions delivered with an iPad result in greater engagement and reduced challenging behavior during the intervention period compared with interventions delivered by teachers and therapists (Fletcher-Watson et al., 2015; Lee et al., 2015; El Zein et al., 2016). These studies did not all demonstrate better learning when the intervention was delivered by the iPad. However, children's *motivation to engage* with learning material should not be overlooked, because motivation processes directly impact knowledge acquisition and transfer (Dweck, 1986). Thus, the attractiveness of these new technologies may be usefully exploited to support better learning outcomes and future research needs to identify how best to achieve this.

Parents can also be enthusiastic about iPads and, in particular, their therapeutic potential due to engagement, which may result in greater use and learning. Clark et al. (2015) found that parents of children with ASD and professionals specializing in ASD both held positive attitudes toward iPad use. The parents in this study reported that 97% of their children used an iPad at a frequency of 4.6 out of 5 days on average, and ~65% of professionals integrated iPads into their practice (e.g., as an intervention or a reward). However, a recent study by Allen and colleagues (Allen A. A. et al., 2015) suggests that parents' positive attitudes toward the iPad are not always enduring. Parents of children who owned, and did not own, an iPad answered questions concerning the potential usefulness of the technology for enhancing their children's communication. Notably, the expectations of parents with children who did *not* own an iPad were significantly more positive than those of parents with children who *had* used an iPad-based AAC. The authors argue that these results indicate “…a conflict between the non-users' illusions and the users' subjective reality regarding the iPad's potential to improve augmentative and alternative communication…” for children with ASD (Allen A. A. et al., 2015, p. 41).

### Future research and the potential of language and communication apps for ASD: a consideration of their design features

The iPad advantage found for engagement and time on task may arise because of the game-like interface of many apps, which successfully promotes these processes, as noted above. However, we must not assume that all apps are equal: specific features may influence the quality of engagement (Kucirkova et al., 2014a), and therefore motivation and learning. Any therapeutic intervention needs to be individualized to meet the needs of a particular child, as the “blanket” materials often implemented do not account for individual differences (Tsatsanis, 2004). It is important to inform the design of any app with expert advice from practitioners and, when possible, to seek input from the children themselves to ensure the content is appropriate for users with ASD (Fletcher-Watson et al., 2016). One reason for the absence of clear advantages for interventions delivered by apps vs. more traditional methods may be to do with a failure to consider and exploit specific features of apps that can benefit learning.

Digital technology affords a unique advantage and opportunity for customization that traditional paper material cannot provide. Apps can either be “closed” or “open”: both are interactive, but only the latter allows the user to change or modify content. For children with ASD, personalization of content to support communication may be critical. Communication apps that use picture based systems (such as PECS; Bondy and Frost, 1994) historically required an individual to print out pre-drawn icons, which were not perceptually similar to real world referents and thus often opaque and difficult to learn. The functionality of tablets with inbuilt cameras can be exploited by apps; actual items can be instantly photographed and included in an individual's picture repertoire in a communication app, so that a child's own objects can now be easily accessed. We have not found any studies that directly compare different functionalities or levels of customization for the same basic app, although some degree of customization currently exists, at least for communication programs such as Proloquo2Go (see Sennott and Bowker, 2009). Samsung's promising “Look at me” app utilizes the camera in digital devices to promote eye contact and allows a customizable experience based upon each child's achievements; empirical testing of its validity is currently underway (The Look at Me Project, 2016)^1^.

Furthermore, in a naturalistic study of TD children's use of different educational apps, Kucirkova et al. (2014a) found that those that allowed personalization by adding photographs, audio, and text comments to create a narrative promoted greater engagement with the task. Similar motivational benefits using a story creation app (“Our Story”) have been reported for children with language and communication difficulties (Kucirkova et al., 2014b; Critten and Kucirkova, 2015). Thus, personalization could be usefully exploited to facilitate engagement.

Apps (for word learning at least) could also be developed to exploit children's word-learning biases. As noted earlier, TD infants show a shape bias when generalizing new labels for objects, whereas children with ASD do not. Apps can be developed to allow fine-grained customization of pictorial features, which could usefully support learning in ASD. For example, it is easier to program an app than to print a book to present multiple differently-colored examples of a target referent rather than a single exemplar. Such features could be used when teaching a new name to highlight the similarity of shape, and foster shape-based generalizations in this population.

We are not suggesting that technology can replace all learning experiences. We note the importance of sensorimotor experiences and attention, and their roles in facilitating word learning in young children (Yu et al., 2008). Further to this, two-dimensional (2-D) representations presented on a screen do not afford manipulation nor provide cues from the caregiver's direction of gaze. However, one of the key features of the tablets on which apps are used is their touch-screen; thus, touch, gesture, and pointing can be supported by the use of tablet hardware (Flewitt et al., 2014) and the apps used to support learning could be designed to enhance this type of sensory interaction to a greater extent than possible with traditional print medium embedded. In addition, the flexibility of an app to manipulate perceptual features of stimuli might be usefully exploited to support learning in children with ASD. Virtual environments can also provide for extra processing time (Southall, 2013) and reduce anxiety associated with face-to-face interactions and thus may be particularly beneficial for children with ASD.

Another way that apps for tablets can be exploited to support learning is scaffolding. Scaffolding refers to the assistance provided to learners on an “as needed basis” that enables them to acquire skills and accomplish tasks that they cannot manage independently (Wood et al., 1976). It might include simplifying the task, at first, providing verbal and visual prompts, and modeling to facilitate success and learning. More support is required when a task (or behavior) is new, and the level of support is gradually reduced as gains in behavior are observed and competence develops. Scaffolding has been shown to be effective in facilitating learning across a wide range of content domains and age and ability ranges (Wood and Wood, 1996). Effective scaffolding might further benefit learning because it could reduce errors made on task, resulting in faster, and more robust learning (Warmington et al., 2013).

Adaptive scaffolds can be embedded into apps to structure and support an individual's learning, just as caregivers and teachers make adjustments during interactions to enable success. Where technology may have one advantage over child-human instructor interactions, is in the ability to program them so that multimodal prompts and supports are readily available to suit the learner's current level. To reflect on our word learning paradigm, an app can be programmed to reinforce and consolidate learning by presenting stimuli in different colors, orientations, etc., more easily than a caregiver or teacher can do so; the latter needs to prepare a range of exemplars in advance and have these all to hand. Further, we can envisage the development of apps that seamlessly move from the highly personalized photographs of objects in an individual's environment, to other photographs, colored pictures, through to black and white line drawings to promote generalization in tune with the learner's performance.

### Recommendations for app use

In light of the ever-growing number of “autism communication apps,” it is increasingly important that parents and practitioners are directed toward software that is most likely to be effective. Boyd et al. (2015) outline five important factors that should be considered when selecting apps for use with children with ASD. Firstly, it is vital to identify apps that are based on scientific principles and/or supported by empirical research (Boone and Higgins, 2007). Helpfully, the website for Autism Speaks, one of the world's leading ASD science and advocacy organizations, lists hundreds of autism-focused software apps and it is possible to filter on the basis of empirical support (Autism Speaks, 2016)^2^. Secondly, it is often favorable to select apps that enable the creation and integration of customized visual supports using the tablet's camera (Sennott and Bowker, 2009). Personalized stimuli of this nature improve the specificity of children's communication, expand opportunities for interaction, and enable caregivers to utilize content that is most likely to facilitate children's symbolic understanding (Hartley and Allen, 2015a,b; Allen M. L. et al., 2015). Thirdly, caregivers should reflect on the motor skills required to effectively engage with a given app. Many children with ASD experience deficits in fine motor skills, and parents should select apps with these difficulties in mind (McNaughton and Light, 2013). Fourthly, it is important to consider the time and resources that are necessary in order to teach children with ASD how to operate and communicate using a given app. While manualized AACs such as PECS have well-established and highly-structured training “stages” (Flippin et al., 2010), there are no standardized guidelines explaining how children with ASD should be taught to use iPads or specific apps, therefore placing emphasis on the caregiver to devise their own strategy (Boyd et al., 2015). Finally, apps should be evaluated on their affordability. Although iPads and their applications are relatively low-cost (in general), they often lack the technical support associated with more conventional AAC devices (McNaughton and Light, 2013). Furthermore, those apps that are supported by empirical research are often expensive (e.g., Proloquo2Go has a download price of £199.99/$249.99). Consequently, some parents may (quite understandably) be tempted by cheaper alternatives that lack empirically-validated efficacy or fail to provide the full range of functionality required by their children.

## Conclusion

Research investigating learning with apps from iPads and electronic media by individuals with ASD is quickly developing as the use of such devices becomes widespread. In terms of strictly promoting spontaneous communication, there does not seem to be an advantage for electronic platforms relative to more traditional picture books. Incorporating the presentation of multiple examples into clinical and educational practices regardless of medium (e.g., the delivery of picture based PECS systems; the development of iPad communication apps) may facilitate understanding in children with ASD that 2-D representations can refer to 3-D objects, leading to improvements in their ability to communicate using pictorial aids.

This review does suggest that digital technology provides one important advantage relative to traditional methods in that it can be easily adapted to accommodate different learning styles and the individual's current knowledge than face-to-face learning: the number of repetitions of material to be learned, the quantity and type of scaffold to aid learning, and the level of difficulty, can all be adjusted automatically based on the learner's response (Akbulut and Cardak, 2012).

Finally, we see an advantage for app-based learning by extending the learning environment. Children do not just learn at school; they learn at home. One advantage of educational apps is that they can provide a seamless transition from school to home, promoting greater learning. This can be critically important for language interventions for children with ASD, where repeated exposure is required.

## Author contributions

MA, CH, and KC contributed to the intellectual contribution and writing of the manuscript.

### Conflict of interest statement

The authors declare that the research was conducted in the absence of any commercial or financial relationships that could be construed as a potential conflict of interest. The reviewer ES and handling Editor declared their shared affiliation, and the handling Editor states that the process nevertheless met the standards of a fair and objective review.

## Footnotes

^1^Retrieved from http://pages.samsung.com/ca/lookatme/English/.

^2^Autism apps. Retrieved May 10, 2016, from https://www.autismspeaks.org/.
